# Multifactor assessment of ovarian cancer reveals immunologically interpretable molecular subtypes with distinct prognoses

**DOI:** 10.3389/fimmu.2023.1326018

**Published:** 2023-12-07

**Authors:** Yaping Guo, Siyu Li, Chentan Li, Li Wang, Wanshan Ning

**Affiliations:** ^1^ Department of Pathophysiology, School of Basic Medical Sciences, Zhengzhou University, Zhengzhou, Henan, China; ^2^ Center for Basic Medical Research, Academy of Medical Sciences, Zhengzhou University, Zhengzhou, Henan, China; ^3^ State Key Laboratory of Esophageal Cancer Prevention and Treatment, Zhengzhou University, Zhengzhou, Henan, China; ^4^ Department of Gynaecology and Obstetrics, Henan Provincial People’s Hospital, Peoples Hospital of Zhengzhou University, School of Clinical Medicine Henan University, Zhengzhou, Henan, China; ^5^ Clinical Medical Research Institute, The First Affiliated Hospital, Xiamen University, Xiamen, Fujian, China

**Keywords:** ovarian cancer, multi-factor, molecular subtypes, support vector machine, diagnostic model

## Abstract

**Background:**

Ovarian cancer (OC) is a highly heterogeneous and malignant gynecological cancer, thereby leading to poor clinical outcomes. The study aims to identify and characterize clinically relevant subtypes in OC and develop a diagnostic model that can precisely stratify OC patients, providing more diagnostic clues for OC patients to access focused therapeutic and preventative strategies.

**Methods:**

Gene expression datasets of OC were retrieved from TCGA and GEO databases. To evaluate immune cell infiltration, the ESTIMATE algorithm was applied. A univariate Cox analysis and the two-sided log-rank test were used to screen OC risk factors. We adopted the ConsensusClusterPlus algorithm to determine OC subtypes. Enrichment analysis based on KEGG and GO was performed to determine enriched pathways of signature genes for each subtype. The machine learning algorithm, support vector machine (SVM) was used to select the feature gene and develop a diagnostic model. A ROC curve was depicted to evaluate the model performance.

**Results:**

A total of 1,273 survival-related genes (SRGs) were firstly determined and used to clarify OC samples into different subtypes based on their different molecular pattern. SRGs were successfully stratified in OC patients into three robust subtypes, designated S-I (Immunoreactive and DNA Damage repair), S-II (Mixed), and S-III (Proliferative and Invasive). S-I had more favorable OS and DFS, whereas S-III had the worst prognosis and was enriched with OC patients at advanced stages. Meanwhile, comprehensive functional analysis highlighted differences in biological pathways: genes associated with immune function and DNA damage repair including *CXCL9*, *CXCL10*, *CXCL11*, *APEX*, *APEX2*, and *RBX1* were enriched in S-I; S-II combined multiple gene signatures including genes associated with metabolism and transcription; and the gene signature of S-III was extensively involved in pathways reflecting malignancies, including many core kinases and transcription factors involved in cancer such as CDK6, ERBB2, JAK1, DAPK1, FOXO1, and RXRA. The SVM model showed superior diagnostic performance with AUC values of 0.922 and 0.901, respectively. Furthermore, a new dataset of the independent cohort could be automatically analyzed by this innovative pipeline and yield similar results.

**Conclusion:**

This study exploited an innovative approach to construct previously unexplored robust subtypes significantly related to different clinical and molecular features for OC and a diagnostic model using SVM to aid in clinical diagnosis and treatment. This investigation also illustrated the importance of targeting innate immune suppression together with DNA damage in OC, offering novel insights for further experimental exploration and clinical trial.

## 1 Introduction

Ovarian cancer (OC) has the highest mortality in gynecological cancers, largely due to the lack of early obvious symptoms and effective screening strategies, resulting in frequent diagnosis at advanced stages (1). The standard of care for OC patients generally encompasses surgery, chemotherapy, and targeted therapy with poly-ADP ribose polymerase inhibitors (PARPi), mainly for patients harboring homologous recombination deficiency (HRD) or a BRCA mutation (1–4). Despite many attempts made to reduce the risks of relapse, the majority of patients suffered recurrences following initial interventions because of high tumor heterogeneity, the immunosuppressive tumor microenvironment (TME), or remaining micrometastases, thereby leading to the poor clinical outcome of OC (5, 6).

Precision medicines have made early successes, which have extensively contributed to refining the classification of complex diseases including cancers and unraveling the underlying driving biomarkers (7–13). Meanwhile, high-throughput muti-omics data or methods constantly exploited also brought cancer into the precision oncology era. Based on the massive information measured, molecular subtyping to guide personalized management of cancer patients has made a big success (8, 14). For example, TCGA study identified three subtypes of colon and rectal cancer based on transcriptome, designated “MSI/CIMP” (microsatellite instability/CpG island methylator phenotype), “Invasive”, and “CIN” (chromosomal instability) (15). Proteome-based stratification of lung adenocarcinoma also revealed three subtypes, designated S-I (environment and metabolism high), S-II (mixed type), and S-III (proliferation and proteasome) (16). The identified subtypes exhibited distinct molecular and clinical features, laying the foundation for more precise diagnosis and treatment in the clinic.

To date, plenty of molecular subtypes of OC have been proposed through molecular profiling of OC patients tissues. In 2008, Tothill et al. applied an unsupervised approach, K-means clustering, to determinate the pioneering molecular subtypes of OC using microarray gene expression profiling (17). They conducted the microarray gene expression profiling on a cohort of 285 serous and endometrioid OC samples originated from the ovary, fallopian tube, and peritoneum, which could be clustered into six optimal subtypes, namely, subtypes C1, C2, C3, C4, C5, and C6. Patients belonging to the C3 and C6 subtypes harbored a distinct molecular signature of lower proliferation marker expression and shared predominantly low-grade or early-stage tumor and thus had better PFS and OS. In contrast, the C1 subtype was characterized with enhanced stromal gene expression but low number of intratumoral T cells and up to 40% of tumors exhibited a lower tumor percentage in contrast to other subtypes, which ultimately resulted in the poorest survival and OS (17). Based on around 1,500 intrinsically variable genes, The Cancer Genome Atlas (TCGA) team implemented non-negative matrix factorization consensus clustering and yielded four subtypes for 489 high-grade serous ovarian adenocarcinomas samples, which were termed as Immunoreactive, Differentiated, Proliferative, and Mesenchymal, based on gene signature in the clusters. The result was also validated using the dataset of Tothill et al. with the same approach. In this perspective, both studies proved that OC patients could be stratified and managed according to accurate molecular signatures and personalized treatment planning based on therapeutic vulnerabilities, which could contribute to achieving precision oncology, rather than be solely dependent on the histologic classification. However, as these OC subtypes do not share obviously distinct prognoses among all the clusters, their application in prognostic evaluation is limited (**Figure S1A**).

**Figure 1:**
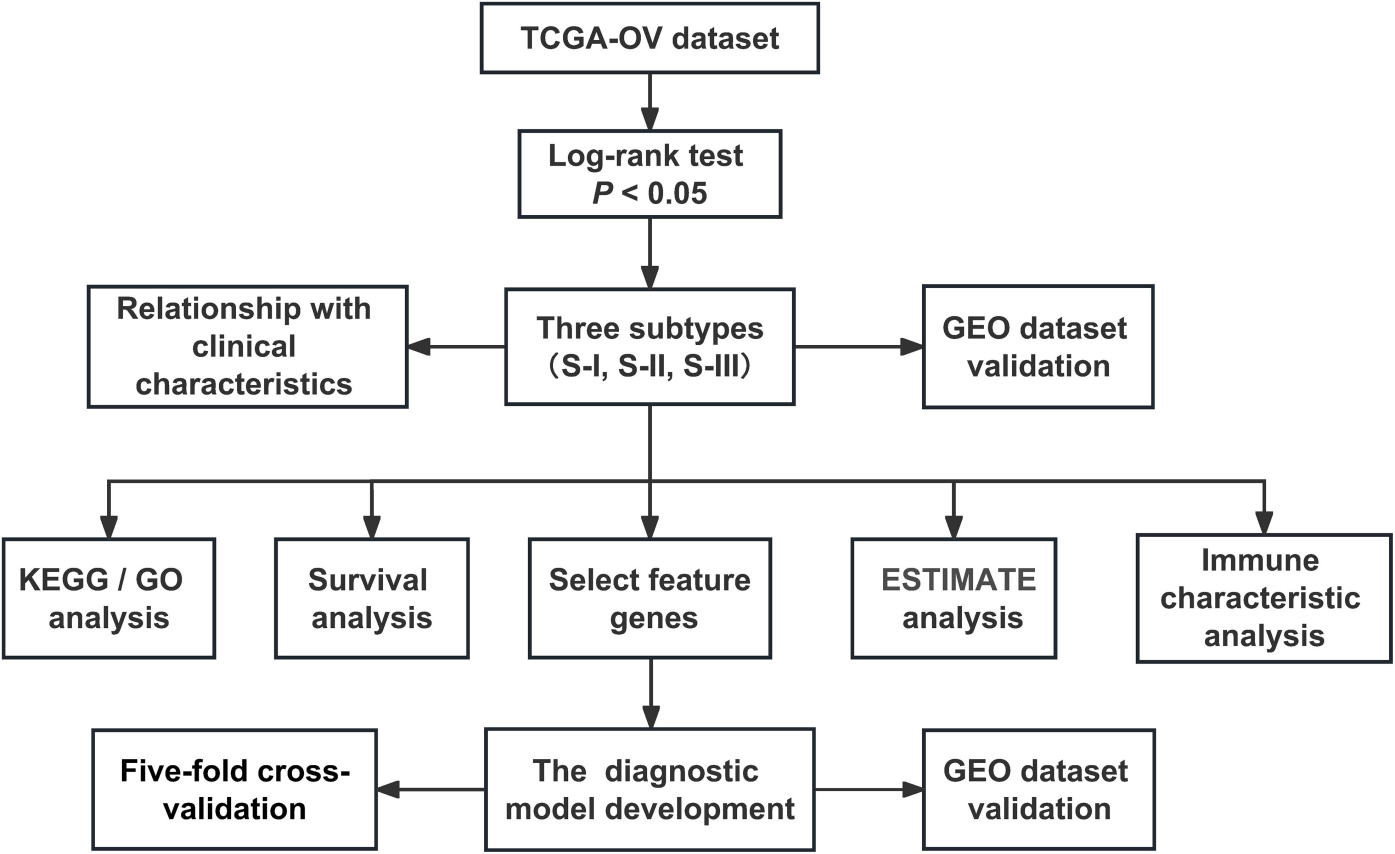
The flowchart of the research design.

In this study, we firstly identified 1,273 SRGs to stratified OC patients into three subgroups, designated “Immunoreactive and DNA damage repair,” “Mixed,” and “Proliferative and Invasive,” based on the different molecular patterns of these SRGs. Kaplan–Meier survival analysis was performed to investigate the prognosis of the three clusters, reflecting that the prognosis of patients in S-I was optimum, where patients in S-I had more favorable OS and DFS than those in S-II and S-III. Notably, a new dataset of the independent cohort could be automatically analyzed by this method and yield similar results. We also found that the clinical stages showed significant differences in the three molecular subtypes. Here, OC patients at a late stage were more enriched with in S-III compared with the other subtypes, which was consistent with the worst prognosis in S-III. Subsequently, our study showed that potentially reprogramming M2-like pro-tumor macrophages into an M1-like antitumor state might facilitate antitumor immunity for OC patients and illustrate synergy of immune microenvironments such as M1 and DNA damage repair molecules might contribute to a favorable prognosis for OC patients. Moreover, we adopted a machine learning algorithm SVM to develop a diagnostic model and the results of the fivefold cross-validation and testing of the independent cohort achieved area under curve (AUC) values of 0.922 and 0.901, respectively, which could precisely stratify OC patients into two subgroups sharing completely different outcomes (**Figure 1**).

## 2 Methods

### 2.1 Data extraction

We downloaded TCGA-OV gene expression dataset from TCGA cohort (https://xenabrowser.net/datapages/?cohort=TCGA%20Pan-Cancer%20(PANCAN)&removeHub=https%3A%2F%2Fxena.treehouse.gi.ucsc.edu%3A443) (**Table S1**).

The GSE26712 expression dataset from the GEO database was downloaded from NCBI (https://www.ncbi.nlm.nih.gov/geo/) (**Table S1**) (18). Data of normal (non-tumor) tissue samples were removed, and OV tissue samples were preserved. The GEO dataset was used for the validation of prognosis-related subtyping, immune characteristics, and predictive model.

Data of immune-related genes were downloaded from the ImmPort database (https://immport.niaid.nih.gov), and duplicates were removed (19).

### 2.2 Data normalization

To ensure the data quality and maximally use the gene expression profile data, mRNA quantified in 100% samples were reserved. The log2-transformed median normalized RSEM counts were used for the following analysis.

### 2.3 Survival analysis

The log-rank test and Cox proportional hazards regression (HR) methods were used to fit survival models for survival analyses between groups (20). To evaluate the prognostic power of each gene, immune characteristics, or molecular subtyping and screen OC risk factors, the two-sided log-rank test was performed for the OC data (P< 0.05), and the Kaplan–Meier survival curve was illustrated by the “survival” R package (survival 3.5-7, https://cran.r-project.org/web/packages/survival/index.html) (21). Finally, we obtained 1,273 SRGs (log-rank *P*< 0.05) from TCGA-OV dataset and 1481 SRGs from the GEO dataset (**Table S2**).

### 2.4 Enrichment analysis

The two-sided hypergeometric test was adopted for the GO- or KEGG-based enrichment analysis of the genes, respectively. Here, we defined the following:

*N* = number of human genes annotated by at least one term
*n* = number of human genes annotated by term *t*
*M* = number of the target gene sets annotated by at least one term
*m* = number of the target gene sets annotated by term *t*

Then, the enrichment ratio (E-ratio) was calculated and the P value was computed with the hypergeometric distribution as below:


$$\text{E}-\text{ratio }=\frac{\frac{m}{M}}{\frac{n}{N}}$$

$$\text{P }=\sum_{m^{\prime }=m}^{n}\frac{\left(\begin{array}{c} M\\ m^{\prime } \end{array}\right)\left(\begin{array}{c} N-M\\ n-m^{\prime } \end{array}\right)}{\left(\begin{array}{c} N\\ n \end{array}\right)},\;\left(E-ratio\;>\;1\right)$$

In this study, only statistically overrepresented GO terms and KEGG pathways were considered (P< 0.05). GO annotation files (released on 09/10/2023) were downloaded from the Gene Ontology Consortium web site (http://www.geneontology.org/) (22). KEGG annotation files (released on 01/01/2021) were downloaded from the webserver of KEGG (http://www.genome.jp/kegg/) (23).

### 2.5 Molecular subtyping analysis

First, the normalization expression values of 1,273 SRGs from TCGA dataset were used for clustering using ConsensusClusterPlus (version 1.64.0, http://bioconductor.org/packages/release/bioc/html/ConsensusClusterPlus.html) package of R (24). The basic parameters were set as follows: *k*-means clustering with up to six clusters (maxK = 6), 1,000-time repetitions (reps = 1,000), resampling 80% of samples (pItem = 0.8), and resampling 80% of proteins (pFeature = 0.8). The number of clustering was determined by the average pairwise consensus matrix within consensus clusters, and by the delta plot of the relative change in the area under the cumulative distribution function (CDF) curve. Because the consensus matrix with *k* = 3 deemed to be a cleanest separation among clusters, and the delta plot showed that there was little increase in area for *k* = 3 compared with *k* = 4, the gene expression data were clustered into three subtypes.

### 2.6 Immune characteristic analysis

We used the “ESTIMATE” package to calculate ESTIMATEScore, ImmuneScore, and StromalScore values in each sample and analyzed the distribution differences of scores in the three subtypes (25). We further used the “CIBERSORT” package to calculate the distribution of the 22 immune cells in each sample (26).

### 2.7 Relationship of subtypes with clinical characteristics

To determine the relationships between subtypes and clinical phenotypes, we analyzed the relationship between each subtype and age, stage, and status and observed the distribution of each subtype.

### 2.8 Molecular characteristics of subtypes

To observe each subtype’s enriched pathway, we first analyzed the differences in gene expression among three subtypes and the highly expressed genes in each subtype were selected. For highly expressed genes of each subtype, we further analyzed the GO- and KEGG-based enrichment analyses of the genes, respectively (**Table S3**).

### 2.9 Relationship of subtypes with immune characteristics

To determine the relationships between subtypes and immune characteristics, the differential abundance distribution of 22 immune cells among three subtypes was calculated using CIBERSORT (**Table S4**).

### 2.10 Correlation between molecular and immune cell characteristics of subtypes

The abundance correlations between mRNA and immune cells were measured using Spearman’s correlation (27). For the abundance correlation, the coefficients of each mRNA and each immune cell were calculated for each tumor sample, respectively.

### 2.11 The machine learning algorithm to predict high- and low-risk subtypes

To predict high- and low-risk subtypes from OC SRGs, we developed the machine learning model with three steps, including feature gene selection, model training, and validation. S-I was taken as the low-risk subtype, whereas S-III was taken as the high-risk subtype. From 149 SRGs identified jointly in TCGA and GEO datasets, feature genes were selected using the Recursive Feature Elimination with Cross-Validation (RFECV) package of python 3.7 with Scikit-learn 0.22.1. For model training, the SVM algorithm was adopted for the combination of these feature genes (28, 29) and fivefold cross-validation was conducted to calculate the AUC value. Finally, we performed model validation using the GEO dataset and calculate the AUC value.

### 2.12 Statistical analysis

The methods of statistical analysis for transcriptomic data analysis, immune characteristic analysis, and clinical characteristic analysis are carefully described in corresponding Methods subsections. Standard two-sided statistical tests used in this study included but are not limited to log-rank test, Spearman’s correlation, Student’s t test, and Kruskal–Wallis test. For categorical variables *vs*. categorical variables, the Student’s t test was used. For categorical variables *vs*. continuous variables, the Kruskal–Wallis test was used to test if any of the differences between the subgroups were statistically significant. For continuous variables *vs*. continuous variables, Spearman correlation was used. The threshold of the P value was set as 0.05.

## 3 Results

### 3.1 Identification of molecular subtypes of OC

**Figure 2:**
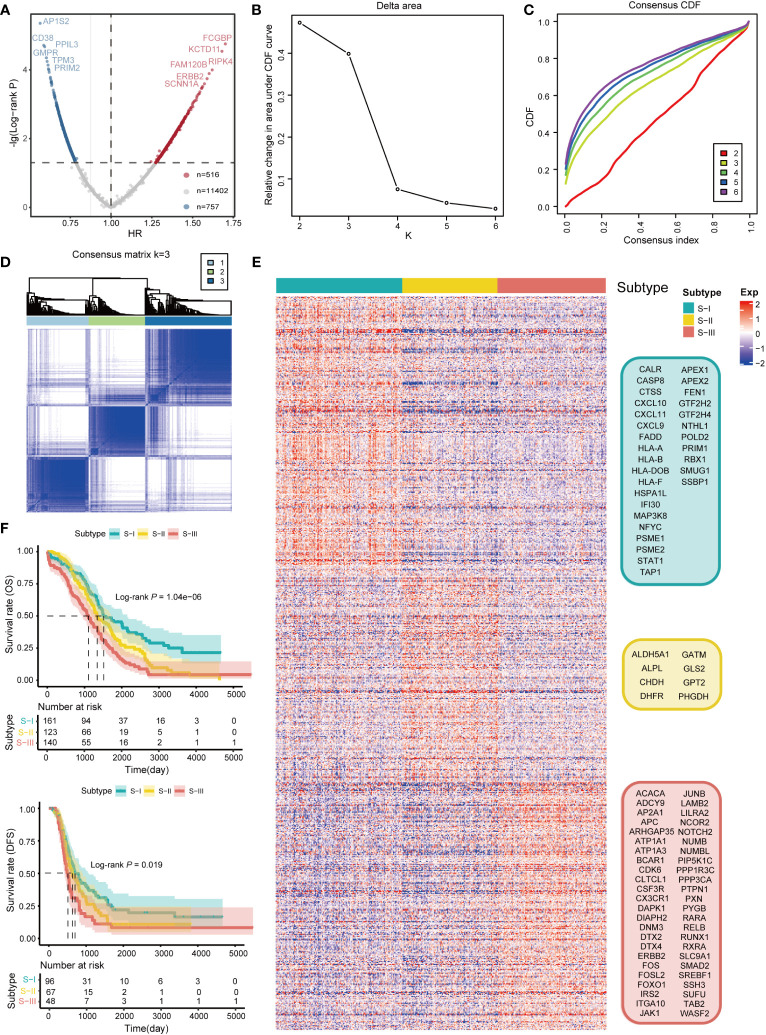
Identification of molecular subtypes of OC. **(A)** Scatterplot showing HR and −lg (log-rank *P*) of all genes (n = 12,675) in TCGA database. The gray dots denote the genes with log-rank *P* value ≥ 0.05 (n = 11,402). In the remaining dots, each red dot denotes an individual gene passing HR >1 (n = 516), and the blue dot denotes an individual gene passing HR<1 (n = 757). Only the first six genes with maximum or minimum HR and log-rank *P*< 0.05 are marked with gene names. **(B)** Line graph of the relative change in area under the CDF curve of clustered samples. **(C)** CDF curve of clustered samples. **(D)** Heatmap of consensus matrix at k = 3. **(E)** Heatmap of normalized gene expression of 1,273 SRGs in all samples. The three subtypes of the sample are shown in different colors. Characteristic gene markers are denoted to the right. **(F)** Kaplan–Meier curves for overall survival (OS) and disease-free survival (DFS) stratified by OC subtypes. Log-rank test is used in **(F)**.

Firstly, a total of 1,273 SRGs were identified using the log-rank test and Cox proportional HR methods, among which 516 genes were high-risk genes (HR >1) and 757 genes were low-risk genes (HR<1) (**Figure 2A**, **Table S2**). Based on 1,273 SRGs, we used the “ConsensusClusterPlus” package with parameters such as the k-means clustering algorithm and 1,000 iterations to perform clustering. Accordingly, we stratified OC patients into three subgroups, designated “S-I, Immunoreactive and DNA damage repair,” “S-II, Mixed,” and “S-III, Proliferative and Invasive,” based on the different molecular patterns of these SRGs **Figures 2B–D**. Subsequently, a heatmap of signature genes for each subtype was depicted to highlight distinct expression profiles. S-I showed high expression of pathway genes related to the immune system and DNA damage repair such as *CXCL9*, *CXCL10*, *CXCL11*, *HLAA*, *HLAB*, *HLAF*, *APEX*, *APEX2*, and *RBX1*. S-II was characterized by genes mainly associated with metabolic and transcription pathways (*GLSL*, *SSDH*, *ALAT2*, *DYR*, *PPBT*, *GATM*, *CHDH*, *SERA, PRPF39, THOC1*), while the gene signature of S-III was extensively involved in pathways reflecting malignancies, including many core kinases and transcription factors involved in cancer such as *CDK6*, *ERBB2*, *JAK1*, *DAPK1*, *FOXO1*, and *RXRA* (**Figure 2E**). In addition, Kaplan–Meier survival analysis reflected that the prognosis of patients in S-I was optimum and patients in S-III had the lowest OS and DFS rates (**Figure 2F**). Next, we conducted separate investigations on the same LUAD dataset from TCGA using both the traditional analysis strategy based on traditional and our subtyping strategy for comparison. Remarkably, we observed significant differences between the two approaches, as the subtypes identified by the former did not exhibit distinct prognostic outcomes (**Figures S1B, C**).

### 3.2 Molecular features of OC subtypes with distinct prognoses

**Figure 3:**
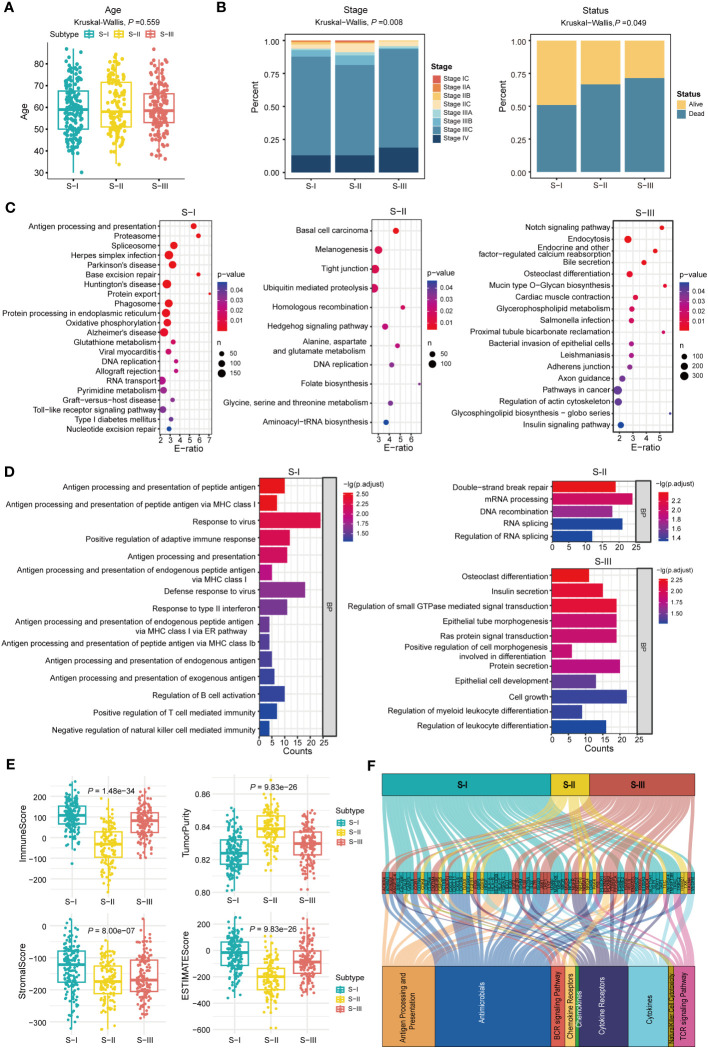
Molecular features of OC subtypes with distinct prognoses. **(A)** The age of all patients in each subtype, P = 0.559 (Kruskal–Wallis test). **(B)** The fractions of clinical stage and status in each subtype, P = 0.008 (stage) and P = 0.049 (status), Kruskal–Wallis test is used in **(B)**. **(C)** KEGG enrichment analysis in each subtype. **(D)** GO-BP enrichment analysis in each subtype. **(E)** ImmuneScore, TumorPurity, StromalScore, and ESTIMATEScore were calculated using the “ESTIMATE” package in R (Kruskal–Wallis test). **(F)** Alluvial diagram establishing associations among molecular subtypes, immune-related genes, and immune pathways.

As shown in **Figures 3A, B**, the molecular subtypes of OC are significantly correlated with clinical stage in OC patients. Here, OC patients at late stages were more enriched with S-III compared with other subtypes, which was consistent with the worst prognosis in S-III, but there was no difference in age among the three molecular subtypes. Furthermore, as shown in **Figure 3C** and **Table S3**, KEGG enrichment analysis revealed that S-I was significantly enriched in pathways related to the immune system and DNA damage repair such as antigen processing and presentation (hsa04612), allograft rejection (hsa05330), graft-versus-host disease (hsa05332), toll-like receptor signaling pathway (hsa04620), base excision repair (hsa03410), DNA replication (hsa03030), and nucleotide excision repair (hsa03420), which was consistent with the better prognosis in S-I. In S-II, the genes were significantly enriched in some pathways related to metabolism, transcription, and so on, including alanine, aspartate, and glutamate metabolism (hsa00250), folate biosynthesis (hsa00790), and aminoacyl-tRNA biosynthesis (hsa00970), suggesting that transcription and metabolic reprogramming might play important roles for sustaining tumorigenesis and survival in this stage. Notably, the folate receptor α (FRα) has been found particularly overexpressed in OC, representing a promising biomarker for OC patients (30). In 2023, Ursula et al. found that mirvetuximab soravtansine (MIRV), an antibody–drug conjugate targeting FRα, showed consistent clinically meaningful antitumor activity and safety for OC patients in targeted therapy (31). S-III was enriched with pathways involved in cancer progression such as pathways in cancer (hsa04330), Notch signaling pathway (hsa04330), osteoclast differentiation (hsa04380), insulin signaling pathway (hsa04910), regulation of actin cytoskeleton (hsa04810), and endocrine and other factor-regulated calcium reabsorption (hsa04961). GO enrichment analysis at biological process (BP) levels showed similar results. For instance, in S-I, the genes were mainly enriched in the immune system, such as antigen processing and presentation of peptide antigen (GO:0048002), positive regulation of adaptive immune response (GO:0002821), response to virus (GO:0009615), and defense response to virus (GO:0051607). In S-II, the genes were related to double-strand break repair (GO:0006302), mRNA processing (GO:0006397), and DNA recombination (GO:0006310). In S-III, GO enrichment showed that genes were involved in the pathway of cancer progression, such as osteoclast differentiation (GO:0030316), insulin secretion (GO:0030073), and Ras protein signal transduction and cell growth (GO:0007265) (**Figure 3D**).

Moreover, ESTIMATEScore, ImmuneScore, StromalScore, and TumorPurity for each subtype were computed using the “ESTIMATE” package to infer tumor purity and stromal and immune cell admixture (**Figures 3E**). The ImmuneScore and StromalScore of S-I were higher than those of S-II and S-III. As for TumorPurity, S-I had the lowest score. The result demonstrated that S-I was enriched with immune cells or factors to regulate the immune responses. Furthermore, the alluvial diagram also indicated that the S-I group harbors the highest count of immune-related genes such as *CXCL10*, *CXCL11*, *CXCL9*, *HLA-A*, *HLA-B*, *HLA-DOB*, *HLA-F*, and *STAT1* (**Figures 3F**).

### 3.3 Characteristics of immune infiltration in subtypes

**Figure 4:**
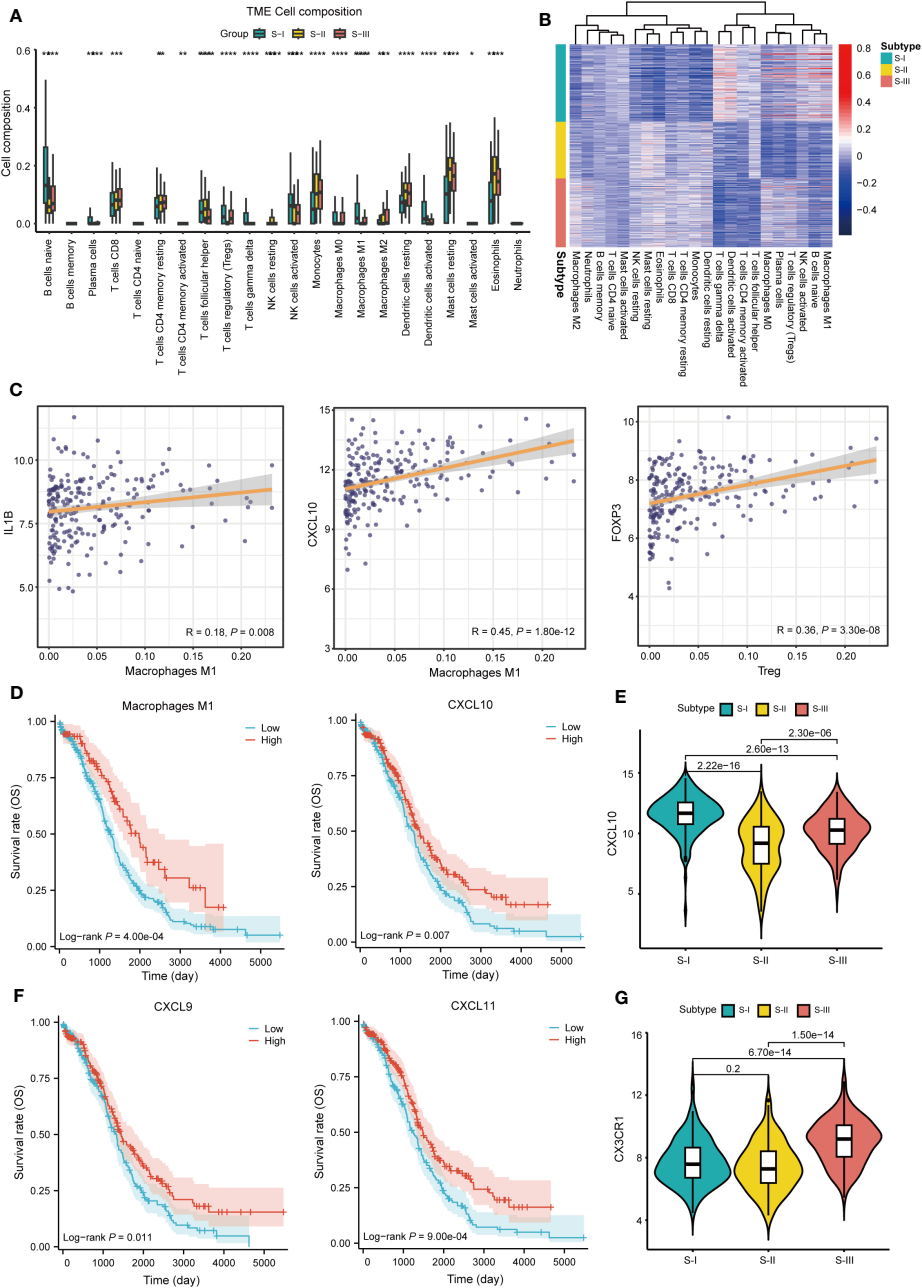
Characteristics of immune infiltration in subtypes. **(A)** The histogram shows the proportion of immune cells among the three subtypes. (*P<0.05,**P<0.01, ***P<0.001, ****P<0.0001). **(B)** Heat map of correlation between gene expression of each subtype and immune cells. **(C)** Correlation display between IL1B, CXCL10 and M1 macrophage, FOXP3, and Treg. **(D)** OS curve of M1 macrophages (log-rank *P* = 4.00e-04) and CXCL10 (log-rank *P* = 0.007). **(E)** Boxplots of CXCL10 expression in each subtype, P = 2.22e-16 (S-I vs. S-II), P = 2.60e-13 (S-I vs. S-III), and P = 2.30e-06 (S-II vs. S-III) Student’s *t*-test is used in **(E)**. **(F)** OS curve of CXCL9 (log-rank *P* = 0.011) and CXCL11 (log-rank *P* = 9.00e-04). **(G)** Boxplots of CX3CR1 expression in each subtype, P = 0.2 (S-I vs. S-II), P = 6.70e-14 (S-I vs. S-III), and P = 1.50e-14 (S-II vs. S-III) Student’ s *t*-test is used in **(G)**.

To elucidate the immune characteristics of each subtype, we conducted immune infiltration analysis using the “CIBERSORT” package. The results showed that compared with S-III, the infiltration level of M1 macrophages in S-I was higher whereas that of M2 macrophages was lower (**Figure 4A**, **Table S4**). Correlation analysis also showed a positive correlation between M1 macrophages and S-I (**Figure 4B**). The correlation analysis also suggested that M1 macrophage-associated genes (*IL1B*, *CXCL10*) showed a significant positive correlation with M1 macrophages (32, 33) and regulatory T cell (Tregs)-associated genes (*FOXP3*) also showed a strong positive correlation with Tregs (**Figure 4C**) (34, 35), confirming the accuracy of our immune infiltration results. Further survival analysis showed that compared with the low-proportion group, the high-proportion group of M1 macrophages exhibited better OS. *CXCL10* also observed a similar trend (**Figure 4D**). The expression level of *CXCL10* was also higher in S-I than in S-II and S-III (**Figure 4E**). In addition, survival analysis was conducted on the immune genes *CXCL9* and *CXCL11*. Patients with high expression levels of *CXCL9* and *CXCL11* also exhibited better OS (**Figure 4F**). By contrast, *CX3CR1* was highly expressed in S-III, which represented inhibitory (CX3CR1^+^) macrophages (36). Evidence also showed that *CX3CR1* was expressed at a lower level in M1 macrophages but at a higher level in M2 macrophages, which can extensively be involved in the migration and survival of tumor cells (**Figure 4G**), confirming the accuracy of subtyping results.

### 3.4 Validation of molecular subtypes in an independent dataset

**Figure 5:**
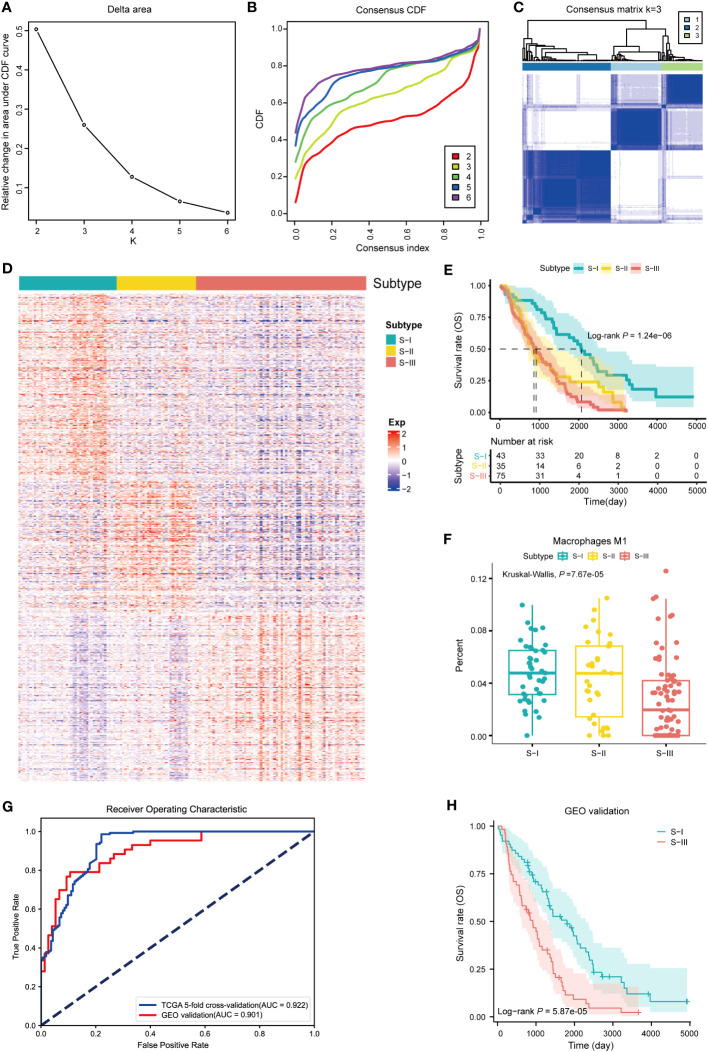
Validation of molecular subtypes and construction of diagnostic models. **(A)** Line graph of the relative change in area under CDF curve of clustered samples. **(B)** CDF curve of clustered samples. **(C)** Heatmap of consensus matrix at k = 3. **(D)** Heatmap of normalized gene expression of 1,481 SRGs in all samples. The three subtypes of the sample are shown in different colors. **(E)** Kaplan–Meier curves for overall survival (OS) stratified by OC subtypes. Log-rank test without adjustment is used in **(E)**. **(F)** The fractions of M1 macrophages in three subtypes, P = 7.67e-05 (Kruskal–Wallis test). **(G)** The AUC curve of diagnostic model with fivefold cross-validation and GEO validation (AUC of GEO validation = 0.901, AUC of fivefold cross-validation = 0.922). **(H)** Kaplan–Meier curve for predicting subtypes of OC based on diagnostic model (log-rank *P* = 5.87e-05).

By GEO database mining, 1,481 SRGs (log-rank *P*< 0.05) were identified through log-rank test between high- and low-expression groups in the independent dataset (**Table S2**). In order to validate the OC molecular subtypes and their prognostic significance, we clustered the independent dataset correlated with OC via the same method. We also obtained similar results (**Figures 5A–C**). Subsequently, a heatmap of distinct expression profiles of each subtype in 1,481 SRGs was depicted (**Figure 5D**). Corresponding OS clinical information was used to generate a Kaplan–Meier survival plot, which is also compatible with the result from a previous study (**Figure 5E**). Furthermore, performing immune characteristic analysis on the verification set, it was found that the percentage of M1 macrophages in S-I and S-II was significantly higher than that in S-III (**Figure 5F**).

### 3.5 Construction of diagnostic models based on SVM

To establish an accurate diagnostic model for distinguishing subtypes with different prognoses, we identified 33 feature genes in TCGA and GEO datasets using RFECV and SVM (**Table S5**). Among them, six genes were overlapped with the ImmPort gene set, namely, *CALR*, *ANGPTL4*, *PSME1*, *TAP1*, *RXRA*, and *CX3CR1*. Using TCGA dataset as the training set and the GEO dataset as the independent validation set, we established a diagnostic model based on the 33 feature genes using SVM. Subsequently, we conducted the fivefold cross-validation on the training set and prediction on the independent validation set. The performance of the diagnostic model was evaluated using the receiver operating characteristic (ROC) curve. In the fivefold cross-validation and independent validation sets, the values of AUC were 0.922 and 0.901 (**Figure 5G**), indicating that the diagnostic model had exceptional classification performance. Further survival analysis of the predicted classification results of the independent validation set showed that patients with S-I had better OS than those with S-III, which was consistent with our validation set clustering results (**Figure 5H**). In order to assure the quality and accuracy of our diagnostic models, we have employed various algorithms, including random forest (RF), penalized logistic regression (PLR), and Bayesian, to construct diagnostic models, which also had exceptional classification performance in common with SVM (**Figures S1D–F**).

## 4 Discussion

OC is the most lethal gynecological tumor and overwhelms worldwide around 207,252 women each year (37). OC encompasses epithelial ovarian, primary peritoneal, or fallopian tube cancer, a phenomenon which represents the extensive heterogeneity in tumor and the TME, largely contributing to patients’ resistance to treatment options, while most of these tumors are sensitive to initial treatment (38). Therapeutic options for OC treatment include cytoreductive surgery, radiotherapy, chemotherapy, and the recent emerging targeted drug therapy (39). For women who carried driver germline mutant in *BRCA1/2*, they were recommended to undergo a risk-reducing salpingo-oophorectomy but should receive hormone replacement therapy if necessary (40). Platinum-based chemotherapy or PARPi including olaparib is the cornerstone for OC treatments, and these treatments applied to patients who carry *HRD* or a *BRCA* mutation were proved to produce durable response and better OS in patients with newly diagnosed advanced OC and a *BRCA* mutation (4). However, drug resistance leading to relapse, widespread intraperitoneal metastasis, and other risk factors are ultimately attributed to the overall high mortality (39). In summary, tumor heterogeneity of OC profoundly compromises patient stratification, prognostic prediction, and personalized treatment, hence leading to the overall poor clinical outcome.

Stratified care has been proved to possess tremendous potential to tailor a more appropriate and effective approach to treatment and improve outcomes in many complex diseases (41–43). In this study, we firstly identified 1,273 SRGs using univariate Cox analysis and successfully stratified OC patients into three subgroups based on the different molecular patterns of these SRGs. To investigate whether the three clusters were related to clinical information, Kaplan–Meier survival analysis was performed, reflecting that patients in S-I had more favorable OS and DFS than those in S-II and S-III. We also found that the clinical stage showed significant differences in the molecular subtypes of OC. Here, the end-stage OC patients were more enriched within S-III compared with the other subtypes, which is consistent with the worst prognosis in S-III. Moreover, the enrichment analysis was conducted based on signature genes belonging to these three clusters, and a deeper characterization of the regulated pathways delineated that genes associated with immune function and DNA damage repair were enriched in S-I whereas signature genes of S-III were extensively involved in pathway reflecting malignancies. For instance, in S-I, the genes were mainly enriched in antigen processing and presentation of endogenous peptide antigen, Toll-like receptor signaling pathway, base excision repair, and nucleotide excision repair. On the contrary, some core kinases and transcription factors involved in cancer such as *CDK6*, *ERBB2*, *JAK1*, *DAPK1*, *FOXO1*, and *RXRA* were highly expressed in S-III. For example, *ERBB2* had been proved to induce transition of adherent cells to non-adherent cells to contribute to peritoneal spread of OC through upregulating *ZEB1* (44) and *CDK6* was found to protect OC cells from death by stabilizing *FOXO3* upon platinum treatment (45). In addition, *FOXO1*, an upstream transcription factor of *SOX2*, participating in cancer stemness had been identified to participate in paclitaxel resistance in OC (17, 46). Moreover, retinoic acid receptor alpha (*RARA*) also highly expressed in S-III was found to mediate DNA polymerase θ expression to promote resistance of PARPi (47). All above evidence explained why patients in S-III tend to have a poor prognosis.

Radiotherapy has been largely abandoned in clinical treatment for OC patients except for palliative care. Although immunotherapy has made big success in many types of cancers such as non-small cell lung cancer (NSCLC), and immune checkpoint inhibitors have been proved to induce significant and sustained responses, OC remains poorly responsive to immunotherapy (48). However, new data indicated that rational combinations of radiotherapy with immunotherapy might impair or eradicate OC, which might suggest that radiation can reprogram the TME to promote antitumor activity in OC (49). In fact, in the TME, many such as tumor-specific expression of CCL5 in conjunction with CXCL9 can enhance lymphocyte infiltration whereas the deficiency of these genes can also promote M2 macrophage differentiation, resulting in immune suppression and poor prognosis. In 2023, *Nikki* also found that tumors especially for end-stage OC patients were relatively devoid of immune cell infiltrates and *CCL5*, which might contribute to a diminished lymphocyte infiltration and shift toward a protumorigenic and immunosuppressive TME (50). In our study, the ESTIMATE algorithm was applied to evaluate immune cell infiltration for each subtype and the results also showed higher immune cell infiltration in S-I compared with S-II and S-III, representing an immunoreactive subtype characterized with higher expression of chemokines such as *CXCL9*, *CXCL10*, and *CXCL11*, indicating a better prognosis, which showed a clear correlation with results of Tothill et al. and TCGA teams (14, 17). Moreover, a deeper characterization of the immune cell infiltration of each of these three subtypes delineated that the infiltration level of M1 macrophages is higher in S-I whereas that of M2 macrophages is lower, when compared with S-III, which also supports that pro-inflammatory M1 macrophages might produce immunostimulatory cytokines such as *CXCL9*, *CXCL10*, and *CXCL11* to maintain its tumoricidal capacity and contribute a better prognosis (39). Additionally, we also found that patients with higher expression of these immunostimulatory cytokines harbor higher OS and a better prognosis, whereas *CX3CR1*, a biomarker of M2 macrophages, also showed different expressions in different clusters especially higher expression in S-III, which might explain why S-I has a better prognosis whereas S-III has the worst prognosis.

To validate the robust subtypes, the same analysis pipeline was applied to a publicly available dataset from GEO, also automatically yielding three clusters and exhibiting a similar molecular expression pattern, survival prognosis, and immune modulation pattern. In order to construct a predictive model to evaluate different prognoses, a machine learning method, SVM, was adopted based on these signature genes. Subsequently, we evaluated the performance of the diagnostic model using the ROC curve. In the fivefold cross-validation and GEO validation, the values of AUC were 0.922 and 0.901 (**Figure 5G**), indicating that the diagnostic model had exceptional classification performance. Further survival analysis of the predicted classification results of the validation set showed that patients in S-I had better OS than S-III (**Figure 5H**), which was consistent with our validation set clustering results.

## 5 Conclusion

Based on bulk transcriptomes, this study exploited a useful approach to construct previously unexplored robust subtypes significantly related to different clinical and molecular features for OC, including *Immunoreactive and DNA damage repair*, *Mixed*, *Proliferative*, and *Invasive*. Additionally, a new dataset can be automatically analyzed by this method and yield similar results. Subsequently, this investigation potentially showed that reprogramming M2-like pro-tumor macrophages into an M1-like antitumor state might facilitate antitumor immunity for OC patients. Our study illustrated that synergy of immune microenvironments such as M1 and DNA damage repair molecules might contribute to a favorable prognosis for OC patients, indicating that this combination might produce therapeutic benefits for OC patients. STING agonists had been proved to reprogram M2-like macrophages into an M1-like state in a macrophage STING-dependent manner and synergizes with PARPi to suppress breast cancer growth. However, a more profound impact on OC remained elusive. Our data illustrated the importance of targeting innate immune suppression together with DNA damage in OC, offering novel insights for future comprehensive research in this field. Moreover, a machine learning algorithm, SVM, was adopted to develop the diagnostic model to aid in clinical identification. Despite of rigorous bioinformatics approaches, this study has limitations including without experimental validation, in spite of being validated in a new dataset from GEO. It is imperative to perform molecular experiments to corroborate the results. Furthermore, large cohorts are needed to deeply test the diagnostic model for OC.

## Data availability statement

The datasets presented in this study can be found in online repositories. The names of the repository/repositories and accession number(s) can be found in the article/**Supplementary Material**.

## Ethics statement

Ethical review and approval was not required for the study on human participants in accordance with the local legislation and institutional requirements. Written informed consent from the patients/participants or patients/participants legal guardian/next of kin was not required to participate in this study in accordance with the national legislation and the institutional requirements.

## Author contributions

YG: Conceptualization, Funding acquisition, Investigation, Writing – original draft, Writing – review & editing. SL: Data curation, Writing – original draft, Writing – review & editing, Formal Analysis, Software, Validation, Visualization. CL: Data curation, Software, Visualization, Writing – original draft, Writing – review & editing. LW: Data curation, Writing – original draft, Writing – review & editing, Investigation. WN: Conceptualization, Funding acquisition, Investigation, Methodology, Supervision, Writing – original draft, Data curation, Formal Analysis, Project administration, Resources, Software, Validation.

## Supplementary material

Supplementary Figure 1
**(A)** Kaplan–Meier curve for previously reported molecular subtypes of OC (log-rank P = 0.417). **(B)** Kaplan–Meier curve for subtypes of LUAD based on traditional subtyping strategy (log-rank P = 0.059). **(C)** Kaplan–Meier curve for subtypes of LUAD based on our subtyping strategy (log-rank P = 5.65e-04). **(D)** The AUC curve of diagnostic model RF-based with fivefold cross-validation and GEO validation (AUC of fivefold cross-validation = 0.940, AUC of GEO validation = 0.889). **(E)** The AUC curve of diagnostic model PLR-based with fivefold cross-validation and GEO validation (AUC of fivefold cross-validation = 0.955, AUC of GEO validation = 0.895). **(F)** The AUC curve of diagnostic model Bayes-based with fivefold cross-validation and GEO validation (AUC of fivefold cross-validation = 0.953, AUC of GEO validation = 0.882).
