# Supplementary figures and images for: Multifactor assessment of ovarian cancer reveals immunologically interpretable molecular subtypes with distinct prognoses
Source: Front Immunol. 2023 Dec 7;14:1326018. doi: 10.3389/fimmu.2023.1326018 (PMC10740166; doi:10.3389/fimmu.2023.1326018)

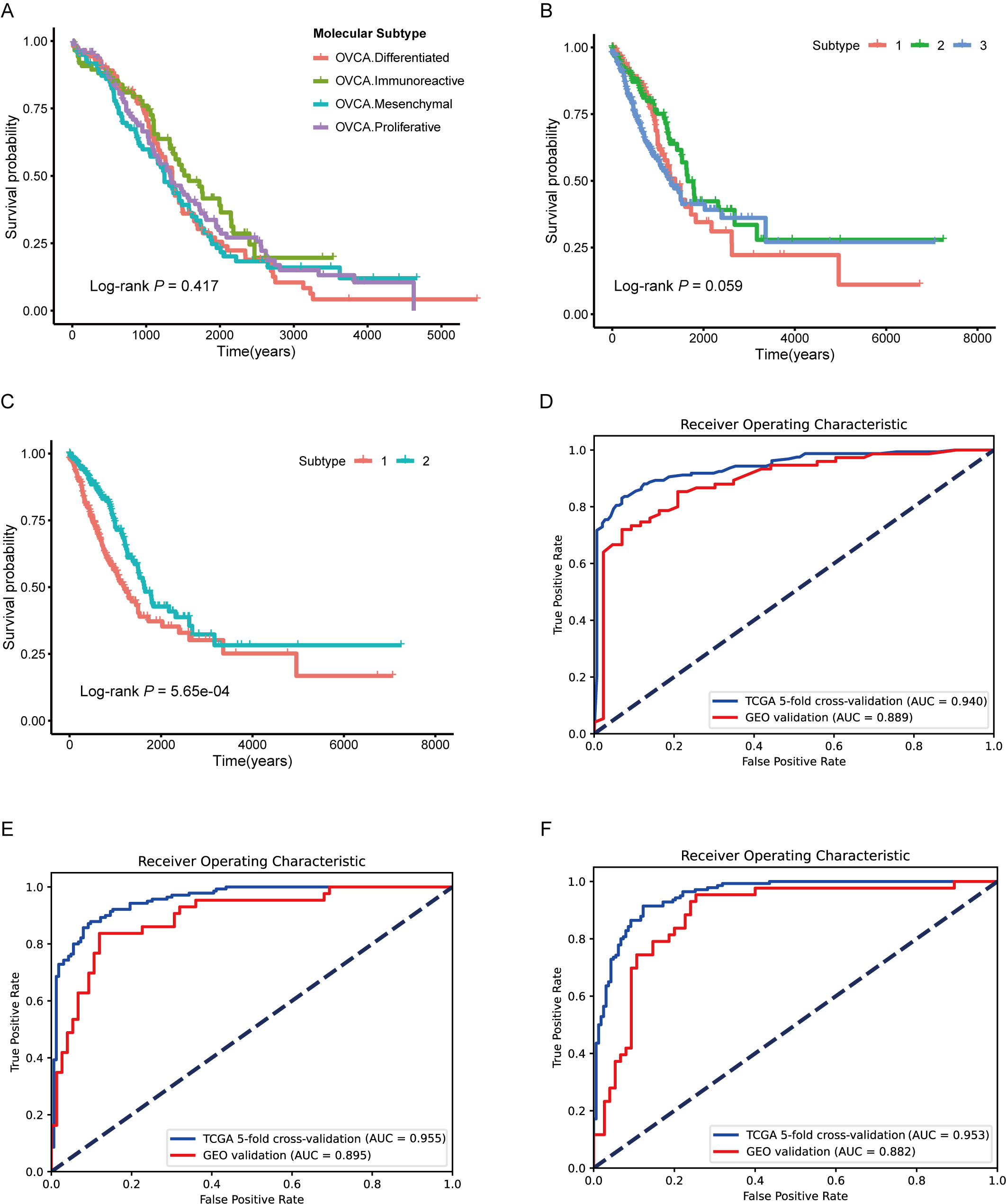

Supplement: Supplementary Figure 1 — (A) Kaplan–Meier curve for previously reported molecular subtypes of OC (log-rank P = 0.417). (B) Kaplan–Meier curve for subtypes of LUAD based on traditional subtyping strategy (log-rank P = 0.059). (C) Kaplan–Meier curve for subtypes of LUAD based on our subtyping strategy (log-rank P = 5.65e-04). (D) The AUC curve of diagnostic model RF-based with fivefold cross-validation and GEO validation (AUC of fivefold cross-validation = 0.940, AUC of GEO validation = 0.889). (E) The AUC curve of diagnostic model PLR-based with fivefold cross-validation and GEO validation (AUC of fivefold cross-validation = 0.955, AUC of GEO validation = 0.895). (F) The AUC curve of diagnostic model Bayes-based with fivefold cross-validation and GEO validation (AUC of fivefold cross-validation = 0.953, AUC of GEO validation = 0.882). [file Image_1.tif]
